# Fixed versus individualized treatment for five common bacterial infectious syndromes: a survey of the perspectives and practices of clinicians

**DOI:** 10.1093/jacamr/dlad087

**Published:** 2023-08-01

**Authors:** Kwadwo Mponponsuo, Ruxandra Pinto, Robert Fowler, Ben Rogers, Nick Daneman

**Affiliations:** Sunnybrook Research Institute, Toronto, Canada; Sunnybrook Research Institute, Toronto, Canada; Department of Critical Care, Sunnybrook Health Sciences Centre, Toronto, Canada; Sunnybrook Research Institute, Toronto, Canada; Department of Critical Care, Sunnybrook Health Sciences Centre, Toronto, Canada; Division of Infectious Diseases, Monash Health, Clayton, VIC, Australia; Sunnybrook Research Institute, Toronto, Canada; Division of Infectious Diseases, Public Health Ontario, Sunnybrook Health Sciences Centre, ICES, Institute of Health Policy Management and Evaluation, University of Toronto, 2075 Bayview Ave, G-wing Room 106, Toronto, ON M4N 3M5, Canada

## Abstract

**Background:**

Traditionally, bacterial infections have been treated with fixed-duration antibiotic courses; however, some have advocated for individualized durations. It is not known which approach currently predominates.

**Methods:**

We conducted a multinational clinical practice survey asking prescribers their approach to treating skin and soft tissue infection (SSTI), community-acquired pneumonia (CAP), pyelonephritis, cholangitis and bloodstream infection (BSI) of an unknown source. The primary outcome was self-reported treatment approach as being fully fixed duration, fixed minimum, fixed maximum, fixed minimum and maximum, or fully individualized durations. Secondary questions explored factors influencing duration of therapy. Multivariable logistic regression with generalized estimating equations was used to examine predictors of use of fully fixed durations.

**Results:**

Among 221 respondents, 170 (76.9%) completed the full survey; infectious diseases physicians accounted for 60.6%. Use of a fully fixed duration was least common for SSTI (8.5%) and more common for CAP (28.3%), BSI (29.9%), cholangitis (35.7%) and pyelonephritis (36.3%). Fully individualized therapy, with no fixed minimum or maximum, was used by only a minority: CAP (4.9%), pyelonephritis (5.0%), cholangitis (9.9%), BSI (13.6%) and SSTI (19.5%). In multivariable analyses, a fully fixed duration approach was more common among Canadian respondents [adjusted OR (aOR) 1.76 (95% CI 1.12–2.76)] and for CAP (aOR 4.25, 95% CI 2.53–7.13), cholangitis (aOR 6.01, 95% CI 3.49–10.36), pyelonephritis (aOR 6.08, 95% CI 3.56–10.39) and BSI (aOR 4.49, 95% CI 2.50–8.09) compared with SSTI.

**Conclusions:**

There is extensive practice heterogeneity in fixed versus individualized treatment; clinical trials would be helpful to compare these approaches.

## Introduction

While IDSA advocates for the implementation of strategies to reduce antibiotic therapy to the shortest effective duration to improve antibiotic usage and reduce adverse events, excess antibiotic treatment duration is common.^1^ A study by Vaughn *et al.*^2^ evaluating antibiotic treatment in hospitalized patients with pneumonia found that 68% of patients received excess antibiotic therapy. Further, excess antibiotic usage was associated with a 5% increase in the odds per day of an antibiotic-associated adverse event.

In his Nobel lecture of the discovery of penicillin, Alexander Fleming stressed the importance of completing the prescribed antibiotic course, warning that stopping prematurely could lead to recurrent and more difficult-to-treat infections for the patient or their family.^3^ Fixed treatment durations have long been the standard for common clinical syndromes, and guidelines recommend either specific treatment durations or ranges of treatment.^4–6^ More recently, many experts have called for the end of fixed antibiotic treatment courses for common bacterial infections, given that ‘patients are put at unnecessary risk from antibiotic resistance when treatment is given for longer than necessary, not when it is stopped early’.^7^ Longer durations expose not only the causative pathogen, but also bystander bacterial flora to more selective pressure.

However, it is not clear whether clinicians commonly recommend individualization of treatment courses or fixed durations of therapy. Further, factors that influence duration of therapy are not well understood. It is conceivable that clinical features such as patient and infection-related factors, pre-existing literature or guidelines, and experience of the treating clinician influence the treatment approach, duration of antibiotics prescribed and when antibiotics are discontinued. We sought to evaluate whether clinicians use a fixed, individualized or a combination of these two treatment approaches for common bacterial infections, and the factors that influence treatment durations.

## Methodology

### Population and general study design

We conducted a multinational clinical practice survey regarding fixed versus individualized treatment recommendations for common bacterial infections. We surveyed self-identified infectious diseases physicians, medical microbiologists, general internists, infectious diseases pharmacists and trainees who were members of the Association of Medical Microbiology and Infectious Diseases (AMMI) in Canada or members of the Australasian Society for Infectious Diseases (ASID) in Australia and New Zealand.

### Survey design

The survey consisted of five common clinical scenarios of bacterial infection: skin and soft tissue infection (SSTI), community-acquired pneumonia (CAP), pyelonephritis, acute cholangitis and bloodstream infection (BSI) of an unknown source. The first question for each scenario was identical and asked whether clinicians use fixed versus individualized treatment approaches. There were five potential answers to the question, which were intended to be mutually exclusive and comprehensive: (1) I use a fixed duration of therapy for most or all patients; (2) I use a fixed minimum duration of therapy, which I extend in certain patients or situations; (3) I use a fixed maximum duration of therapy, which I reduce in certain patients or situations; (4) I use a range of durations with a fixed maximum and minimum, and vary within that range according to patient or situation; (5) I have no fixed minimum or maximum duration, and treat in a manner entirely individualized to the patient or situation.

A fixed duration of therapy was defined as a specific number of days of treatment that a patient would receive for a given infectious syndrome without variability in the days of treatment provided. Fixed minimum duration corresponded to the minimum number of treatment days a patient may receive for a given infectious syndrome with the possibility of increasing the number of days, i.e. *at least* a certain number of days of treatment. Conversely, a fixed maximum duration of therapy denoted the maximum number of treatment days provided with the possibility of reducing the number of days, i.e. *at most* a certain number of treatment days. Exploratory questions evaluated clinical factors such as patient, pathogen, clinical and biochemical factors that may influence therapy; these responses were not mutually exclusive—a prescriber could check any or all factors that would influence prescribing. Terminal questions explored the general perspectives of clinicians regarding fixed versus individualized treatment durations. The full questionnaire is provided in the Supplementary data, available at *JAC-AMR*. Online pilot testing was conducted on 10 specialists in Canada to evaluate the survey flow and ease of administration.^8^ The same individuals were also asked to respond to a clinical sensibility tool to confirm that items included were clear and in keeping with routine clinical practice.

### Survey administration

Members of AMMI Canada and ASID were asked to complete an online survey voluntarily and anonymously via Qualtrics XM. Individuals received an initial invitation to participate, with e-mail reminders sent 2, 4 and 6 weeks later; the surveys were initiated in Canada in March 2022, and in Australia/New Zealand in June 2022. To increase Canadian participant uptake, we directly contacted members of infectious diseases divisions across university sites in Eastern and Western Canada.

### Statistical analysis

Descriptive analyses with summary statistics were used to summarize responses, primarily with proportions and graphical bar plots. Treatment duration approaches were compared across groups based on respondent characteristics using the chi-squared test of proportions. A multivariable logistic regression model was used to compare the syndrome scenarios of clinicians recommending fixed treatment duration as the dependent variable (compared with responses of fully or partially individualized treatment) using a generalized estimating equations model to account for clustering of scenarios within clinicians and adjusting for location (Canada versus Australia/New Zealand), practice setting (academic versus non-academic) and clinical scenario using SSTI as the reference category; these variables were chosen *a priori* based on clinical relevance. In a *post hoc* analysis, a univariable logistic regression model was created with fixed duration of treatment (as compared with responses of fully or partially individualized treatment) as the dependent variable. All statistical analyses were conducted in Microsoft Excel version 16.66 and SAS version 9.4 (SAS Institute Inc., Cary, NC, USA).

## Results

### Participants

Our survey response rate from AMMI Canada was 105/634 (16.6%); the response rate from ASID was 103/883 (11.7%). A total of 221 respondents consented to participate in the survey, of whom 170/221 (76.9%) completed the survey in full and 177/221 (80.1%) answered the primary question indicating their approach to duration of therapy for each clinical scenario. Infectious diseases physicians accounted for 60.6% of respondents and 75% of survey participants practiced in an academic centre. Respondents were predominantly from Canada (48.6%) and Australia/New Zealand (45.7%) (Table 1).

**Table 1.** Baseline demographic information of respondents

	*n* (%)
Clinical specialty	
Infectious diseases physician	126 (60.6)
Infectious diseases physician + medical microbiologist	32 (15.4)
Medical microbiologist	5 (2.4)
Infectious diseases ± medical microbiology trainee	22 (10.6)
Medical microbiology trainee	1 (0.5)
General internal medicine physician	5 (2.4)
General internal medicine trainee	2 (1.0)
Infectious diseases pharmacist	6 (2.9)
Other	9 (4.3)
Geographical location of respondent	
Canada	101 (48.6)
Australia	95 (45.7)
New Zealand	8 (3.9)
Other	4 (1.9)
Location of practice of respondents	
Academic centre	156 (75.0)
Community centre	31 (14.9)
Private practice	8 (3.9)
Other	13 (6.3)

### Fixed versus individualized approaches to duration of therapy

**Figure 1.**
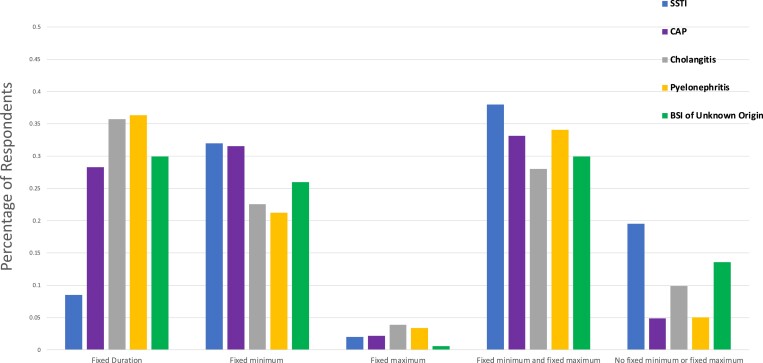
Approach to duration of therapy for each clinical scenario.

There was extensive variation in approaches to durations of therapy, regardless of the clinical scenario (Figure 1). Use of a fully ‘fixed duration’ was least common for SSTI (*n* = 17; 8.5%) and more common for CAP (*n* = 52; 28.3%), BSI of unknown source (*n* = 53; 29.9%), acute cholangitis (*n* = 65; 35.7%) and pyelonephritis (*n* = 65; 36.3%). The least common approach was the ‘fixed maximum’ with less than 5.0% of respondents choosing this option for any scenario. Completely individualized therapy, with ‘no fixed minimum or maximum’ was used by only a minority of respondents; it was least common for CAP (*n* = 9; 4.9%) and pyelonephritis (*n* = 9; 5.0%) and more common for acute cholangitis (*n* = 18; 9.9%), BSI of unknown source (*n* = 24; 13.6%) and SSTI (*n* = 39; 19.5%).

In the multivariable analyses (Table 2), there was higher usage of a completely fixed duration approach for CAP [adjusted OR (aOR) 4.25, 95% CI 2.53-7.13, acute cholangitis (aOR 6.01, 95% CI 3.49-10.36), pyelonephritis (aOR 6.08, 95% CI 3.56-10.39) and BSI of unknown source (aOR 4.49, 95% CI 2.50-8.09) when compared with SSTI. Canadian respondents (compared with other countries) were significantly associated with use of a completely fixed duration [aOR 1.76, 95% CI 1.12-2.76), *P* = 0.01]. Finally, academic practice settings (compared with non-academic) were not associated with use of a fixed duration approach [aOR 0.79, 95% CI 0.47-1.31) *P* = 0.36]. Results of the univariable analyses can be found in Table S1.

**Table 2.** Multivariable analysis examining respondent and infection characteristics associated with use of a completely fixed duration of treatment approach compared with partial or fully individualized treatment approaches

Variable	Fixed duration of treatment approach OR (95% CI)	*P* value
Infectious syndrome		
SSTI	ref	ref
CAP	4.25 (2.53–7.13)	<0.0001
Acute cholangitis	6.01 (3.49–10.36)	<0.0001
Pyelonephritis	6.08 (3.56–10.39)	<0.0001
BSI of unknown origin	4.49 (2.50–8.09)	<0.0001
Country of respondent		
New Zealand and Australia	ref	ref
Canada	1.76 (1.12–2.76)	0.01
Location of practice		
Non-academic	ref	ref
Academic	0.79 (0.47–1.31)	0.36

### Number of days of treatment recommended

**Figure 2.**
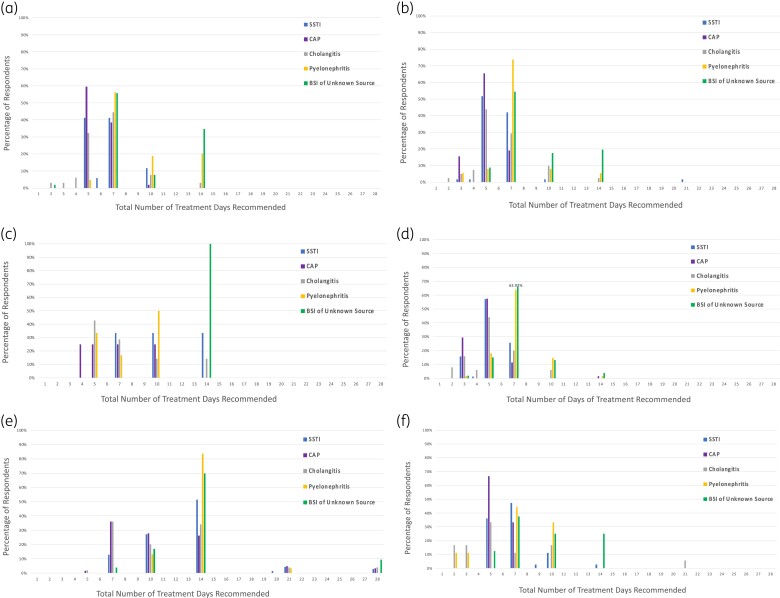
(a) Total numbers of days of treatment provided by those utilizing the fixed duration of therapy approach. (b) Total numbers of days of treatment provided by those utilizing the fixed minimum duration of therapy approach. (c) Total numbers of days of treatment provided by those utilizing the fixed maximum duration of therapy approach. (d) Minimum numbers of days of treatment provided by those utilizing the fixed minimum and fixed maximum duration of therapy approach. (e) Maximum numbers of days of treatment provided by those utilizing the fixed minimum and fixed maximum duration of therapy approach. (f) Most common numbers of days of treatment provided by those with no fixed or fixed minimum or maximum duration of therapy approach.

Regardless of the approach or clinical scenario, 5, 7 and 14 days of treatment were the most commonly recommended durations of therapy (Figure 2a–f). Five or 7 days was the most common duration recommended for cases of SSTI, CAP and cholangitis, regardless of the approach utilized. In cases of pyelonephritis and BSI of unknown source, respondents favoured 7 or 14 days of therapy.

### Factors that influence duration of therapy

**Figure 3.**
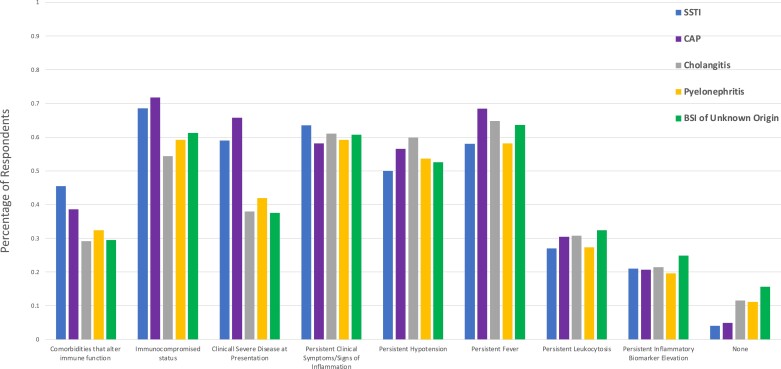
Clinical and biochemical factors that would result in increased duration of therapy based on infectious syndrome.

Across all clinical scenarios, immunocompromised status, persistent clinical signs and symptoms of inflammation and persistent fever were commonly chosen as reasons for extension of the duration of therapy. Clinically severe disease at presentation led to an increase in the duration of therapy for SSTI (59.0%) and CAP (65.8%) but had less of an effect on respondent response to the other scenarios (Figure 3). Further, persistent leucocytosis and inflammatory biomarker elevation had little influence on the duration of therapy recommended for all scenarios (Figure 3).

### Definition and characterization of ‘Day 1’ of therapy

Survey participants were asked to characterize how they determine the ‘first day of therapy’ in cases of cholangitis and BSI of unknown source. Nearly 50% of participants identified the day of source control as the first day of therapy in cases of cholangitis, followed by the first day of receipt of effective antibiotics at 30.9% (Figure S1a). The inverse was elicited in the BSI of unknown source scenario where 53.8% of respondents characterized the first day of therapy as the first day of receipt of effective antibiotics, followed by 26.6% who characterized the first day of therapy as the day of source control if this was needed (Figure S1b). The first day of clinical stability had little to no impact on defining start date.

### Modification of duration of therapy based on pathogen characteristics

In the pyelonephritis scenario, survey participants were asked if their durations of therapy would be altered if the pathogen in question were classified as having an ESBL or being resistant to carbapenems. In both instances, there was a slight incremental increase in the duration of therapy recommended. A similar pattern was observed for the case of BSI of unknown source (data not shown).

## Discussion

We conducted a multinational survey of clinicians regarding the use of fixed versus individualized treatment approaches for five of the most common bacterial infectious syndromes. Fully individualized treatment durations were used by only a small minority of prescribers and were particularly uncommon for CAP and pyelonephritis. There was variability in the duration of therapy approaches, with respondents mainly favouring a completely fixed duration approach, a fixed minimum approach or a fixed minimum and maximum approach. The fully fixed duration approach is more common among Canadian prescribers, and more commonly utilized for CAP, pyelonephritis, cholangitis and bacteraemia of unknown source than for SSTI. Regardless of the approach and clinical scenario, respondents often chose 5, 7 or 14 days of therapy as the recommended number of treatment days.

There have been increasing calls for a precision-medicine approach to antibiotic treatment, giving each patient a tailored antibiotic duration that is exactly as long as needed to cure their individual infection.^7^ However, our multinational survey indicates that this approach remains quite uncommon. Fully individualized treatments, with no minimum or maximum, are used for only 5% of patients with CAP and pyelonephritis and only 10%–20% of patients with other common syndromes. A fully individualized treatment approach may be ideal in theory, but much more challenging to implement in actual clinical practice. First, individualizing treatment requires serial reassessment of a patient’s progress through the trajectory of treatment, and this may be difficult to accomplish—particularly in the outpatient setting. Even when clinical reassessment is feasible, tests of cure are lacking for most infectious disease syndromes, and clinical signs and symptoms of inflammation can persist beyond cure of the inciting pathogen.^9,10^ Last, the minimum duration of therapy required to fully treat common bacterial infections remains unknown. In general, studies tend to work in descending durations and non-inferiority. For example, pyelonephritis has typically been treated for 10 to 14 days, with emerging evidence that a shorter duration such as 7 days is equally effective.^11^ However, the minimum duration of therapy required to treat pyelonephritis currently remains unknown. All of these factors combined make it difficult to implement an individualized approach to treatment.

A fully fixed duration of treatment was much more common than a fully individualized treatment duration recommendation for most of the clinical scenarios. A high usage of completely fixed duration of therapy for common syndromes like CAP and pyelonephritis may reflect the influence of existing clinical guidelines, which provide recommendations for treatment duration for these conditions.^4,6^ A 2018 systematic review on the efficacy of short courses of antibiotics in CAP suggested that short treatment durations were equally as effective as longer courses,^12^ and so the American Thoracic Society (ATS) guidelines recommend no less than 5 days of treatment for CAP.^4^ The pyelonephritis guidelines recommend 7 days of fluoroquinolone treatment, 14 days of trimethoprim/sulfamethoxazole treatment, or 10–14 days of β-lactam treatments.

While clinical practice guidelines have also been published for SSTI, the relatively lower usage of a completely fixed approach in this scenario suggests that clinicians’ perceived ability to re-evaluate clinical signs and symptoms following therapy also influenced the treatment approach and number of days of therapy recommended.^5^ However, it is important to note that persistent signs and symptoms of soft tissue inflammation, such as persistent erythema, swelling or pain, following treatment of SSTI may not reflect the presence of viable bacteria at the site of infection, just as cough and fatigue may not represent viable lung bacteria following treatment of CAP.^10,13–15^ Our exploratory questions revealed that persistent clinical signs and symptoms of inflammation and persistent fever were among the most commonly chosen reasons for extension of the duration of therapy. These findings are similar to prior studies evaluating clinician perspectives on BSIs.^16^ Usage of these parameters should be done with caution to reduce the paradoxical risk of increasing durations of antibiotic therapy when trying to individualize treatments. An interesting finding was the higher odds of a completely fixed duration approach for Canadian respondents compared with other countries. Reasons for this are not immediately clear but may reflect differences in training, clinical practice guidelines and/or local practice variations.

Our study has several limitations to consider. First, the survey was only distributed to clinicians practising in Canada, Australia and New Zealand and does not capture the perspectives of clinicians outside of these regions. Second, invitations to participate were sent via Canadian, Australian and New Zealand infectious diseases and medical microbiology professional societies, and as is typical for surveys administered through professional societies, the overall response rates in our survey was low,^16–18^ and it is not possible to track the characteristics of respondents versus non-respondents to evaluate representativeness of our study population. However, most respondents were practising clinicians (less than 13% of respondents were trainees), increasing the reflection of responses on practice patterns. The survey also relied on self-reported answers, without validation or chart review of the actual prescribing practices of respondents.

Despite these limitations, our study has multiple strengths. To our knowledge, it is the first study conducted evaluating fixed versus individualized approaches to therapy for common bacterial infections. Respondents were diverse and included infectious diseases physicians and pharmacists, medical microbiologists, internal medicine specialists and trainees. Further, the survey was distributed in multiple countries, capturing the variability of practice patterns in different geographical regions. Lastly, the study highlighted the heterogeneity in clinical approaches across clinical syndromes.

### Conclusions

This multinational survey highlighted extensive practice heterogeneity in fixed versus individualized treatment approaches, durations of therapy and influential clinical factors for five common bacterial infectious syndromes. Fully individualized therapy may potentially be an ideal approach to maximize the benefits and minimize the harms of antimicrobials but remains uncommon in clinical practice. Syndromes with established clinical guidelines were more likely to have a fully fixed duration of therapy approach, while those in which clinical reassessment is common following initiation of treatment, were less likely to have a fully fixed approach. Clinical trials would be helpful to compare fixed versus individualized treatment approaches to further advance management of patients with these common infections.

## Supplementary Material

dlad087_Supplementary_Data

## Supplementary data

Survey questions, case scenarios, Table S1 and Figure S1 are available as Supplementary data at *JAC-AMR* Online.
