# Supplementary material for: Fixed versus individualized treatment for five common bacterial infectious syndromes: a survey of the perspectives and practices of clinicians
Source: JAC Antimicrob Resist. 2023 Aug 1;5(4):dlad087. doi: 10.1093/jacamr/dlad087 (PMC10391701; doi:10.1093/jacamr/dlad087)
Supplement: dlad087_Supplementary_Data [file dlad087_supplementary_data.docx]

Supplemental Material

| Survey Questions | Page 2 |
| --- | --- |
| Univariable Analyses | Page 15 |
| Characterization of Day 1 of Therapy | Page 18 |

**Survey Questions**

1. Please indicate your medical specialty
   1. Infectious Diseases Physician
   2. Infectious Diseases Physician + Medical Microbiologist
   3. Medical Microbiologist
   4. Infectious Diseases +/- Medical Microbiology Trainee
   5. Medical Microbiology Trainee
   6. General Internal Medicine Physician
   7. General Internal Medicine Trainee
   8. Other, please specify ________
2. What country do you currently practice in?
   1. Canada
   2. Australia
   3. New Zealand
   4. Other, please specific _______
3. Where is your principal location of practice?
   1. Academic center
   2. Community center
   3. Other, please specify: ____
4. For those in independent clinical practice, how many years have you been practicing independently for?
   1. Still in training
   2. 0 – 5 years
   3. 6 – 10 years
   4. 11 - 15 years
   5. >15 years

**Cases**

1. Cellulitis
2. Pneumonia
3. Acute cholangitis
4. Pyelonephritis
5. Bacteremia of unknown source

**Case Scenario #1**

You are seeing a 54-year-old otherwise healthy male, who was admitted to the hospital 48 hours ago for antibiotics for his first episode of acute left leg cellulitis. He has been started on appropriate therapy.

1a. What is your preferred approach to duration of therapy, for lower limb cellulitis?

1. I use a fixed duration of therapy for most or all patients
2. I use a range of durations with a fixed maximum and minimum, and vary within that range according to patient or situation
3. I use a fixed minimum duration of therapy, which I extend in certain patients or situations (with no fixed maximum)
4. I use a fixed maximum duration of therapy, which I reduce in certain patients or situations (with no fixed minimum)
5. I have no fixed minimum or maximum duration, and treat in a manner entirely individualized to the patient or situation

The following question will vary depending on the answer provided in 1a.

If the respondent chooses “a” in response to 1a, then he/she is asked:

1b: What is the usual fixed duration of therapy you recommend for most or all patients?

Numbered selection from dropdown menu (1 – 28 days)

If the respondent chooses “b” in response to 1a, then he/she is asked:

1b: What is the fixed minimum and maximum duration of therapy you recommend for patients?

2 numbered selections from dropdown menu (1 – 28 days)

If the respondent chooses “c” in response to 1a, then he/she is asked:

1b: What is the fixed minimum duration of therapy you recommend for patients?

Numbered selection from dropdown menu (1 – 28 days)

If the respondent chooses “d” in response to 1a, then he/she is asked:

1b: What is the fixed maximum duration of therapy you recommend for patients?

Numbered selection from dropdown menu (1 – 28 days)

If the respondent chooses “e” in response to 1a, then he/she is asked:

1b: What is the most common duration of therapy you recommend for patients?

Numbered selection from dropdown menu (1 – 28 days)

1c. Would any of the following factors, if present, cause you to increase the duration of therapy proposed? (Check all that apply)

- 1. Comorbidities that alter immune function (e.g., Diabetes mellitus, chronic liver disease, chronic kidney disease)
  2. Immunocompromised status (e.g. hematological malignancy, recent solid organ or hematopoietic stem cell transplantation, or receipt of immunosuppressive medications)
  3. Clinically severe disease at presentation
  4. Persistent clinical symptoms/signs of inflammation at the site of infection
  5. Persistent hypotension
  6. Persistent fever
  7. Persistent leukocytosis
  8. Persistent biomarker of inflammation elevation (e.g., CRP, PCT, ESR)
  9. None of the factors would result in increases to the duration of therapy proposed

**Case Scenario #2**

You are seeing a 60-year-old otherwise healthy female who was admitted for community-acquired pneumonia. She has been started on appropriate therapy.

2a. What is your preferred approach to duration of therapy, for pneumonia?

1. I use a fixed duration of therapy for most or all patients
2. I use a range of durations with a fixed maximum and minimum, and vary within that range according to patient or situation
3. I use a fixed minimum duration of therapy, which I extend in certain patients or situations (with no fixed maximum)
4. I use a fixed maximum duration of therapy, which I reduce in certain patients or situations (with no fixed minimum)
5. I have no fixed minimum or maximum duration, and treat in a manner entirely individualized to the patient or situation

The following question (2b) will vary depending on the answer provided in 2a.

If the respondent chooses “a” in response to 2a, then he/she is asked:

2b: What is the usual fixed duration of therapy you recommend for most or all patients?

Numbered selection from dropdown menu (1 – 28 days)

If the respondent chooses “b” in response to 2a, then he/she is asked:

2b: What is the fixed minimum and maximum duration of therapy you recommend for patients?

2 numbered selections from dropdown menu (1 – 28 days)

If the respondent chooses “c” in response to 2a, then he/she is asked:

2b: What is the fixed minimum duration of therapy you recommend for patients?

Numbered selection from dropdown menu (1 – 28 days)

If the respondent chooses “d” in response to 2a, then he/she is asked:

2b: What is the fixed maximum duration of therapy you recommend for patients?

Numbered selection from dropdown menu (1 – 28 days)

If the respondent chooses “e” in response to 2a, then he/she is asked:

2b: What is the most common duration of therapy you recommend for patients?

Numbered selection from dropdown menu (1 – 28 days)

2c. Would any of the following factors, if present, cause you to increase the duration of therapy proposed? (Check all that apply)

1. Comorbidities that alter immune function (e.g., Diabetes mellitus, chronic liver disease, chronic kidney disease)
2. Immunocompromised status (e.g. hematological malignancy, recent solid organ or hematopoietic stem cell transplantation, or receipt of immunosuppressive medications)
3. Clinically severe disease at presentation
4. Persistent clinical symptoms/signs of inflammation at the site of infection
5. Persistent hypotension
6. Persistent fever
7. Persistent leukocytosis
8. Persistent biomarker of inflammation elevation (e.g., CRP, PCT, ESR)
9. None of the factors would result in increases to the duration of therapy proposed

Case Scenario #3

You are seeing a 45-year-old previously healthy male who was admitted with acute cholangitis. He underwent ERCP with sphincterotomy for source control.

3a. What is your preferred approach to duration of therapy, for cholangitis/biliary disease?

1. I use a fixed duration of therapy for most or all patients
2. I use a range of durations with a fixed maximum and minimum, and vary within that range according to patient or situation
3. I use a fixed minimum duration of therapy, which I extend in certain patients or situations (with no fixed maximum)
4. I use a fixed maximum duration of therapy, which I reduce in certain patients or situations (with no fixed minimum)
5. I have no fixed minimum or maximum duration, and treat in a manner entirely individualized to the patient or situation

The following question (3b) will vary depending on the answer provided in 5a.

If the respondent chooses “a” in response to 3a, then he/she is asked:

3b: What is the usual fixed duration of therapy you recommend for most or all patients?

Numbered selection from dropdown menu (1 – 28 days)

If the respondent chooses “b” in response to 3a, then he/she is asked:

3b: What is the fixed minimum and maximum duration of therapy you recommend for patients?

2 numbered selections from dropdown menu (1 – 28 days)

If the respondent chooses “c” in response to 3a, then he/she is asked:

3b: What is the fixed minimum duration of therapy you recommend for patients?

Numbered selection from dropdown menu (1 – 28 days)

If the respondent chooses “d” in response to 3a, then he/she is asked:

3b: What is the fixed maximum duration of therapy you recommend for patients?

Numbered selection from dropdown menu (1 – 28 days)

If the respondent chooses “e” in response to 3a, then he/she is asked:

3b: What is the most common duration of therapy you recommend for patients?

Numbered selection from dropdown menu (1 – 28 days)

3c. Would any of the following factors, if present, cause you to increase the duration of therapy proposed? (Check all that apply)

1. Comorbidities that alter immune function (e.g., Diabetes mellitus, chronic liver disease, chronic kidney disease)
2. Immunocompromised status (e.g. hematological malignancy, recent solid organ or hematopoietic stem cell transplantation, or receipt of immunosuppressive medications)
3. Clinically severe disease at presentation
4. Persistent clinical symptoms/signs of inflammation at the site of infection
5. Persistent hypotension
6. Persistent fever
7. Persistent leukocytosis
8. Persistent biomarker of inflammation elevation (e.g., CRP, PCT, ESR)
9. None of the factors would result in increases to the duration of therapy proposed

3d. When recommending a duration of therapy, how do you define “Day 1” of treatment?

1. I start “day 1” of therapy from the first day of receipt of antibiotics
2. I start “day 1” of therapy from the first day of the definitive antibiotic therapy provided
3. I start “day 1” of therapy from the first day of negative blood cultures
4. I start “day 1” of therapy from the day of source control
5. I start “day 1” of therapy from the day of clinical stability
6. Other, please specify: _______

Case Scenario #4

You are seeing a 63-year-old female with left-sided pyelonephritis. Urine cultures are positive for *Proteus mirabilis*.

4a. What is your preferred approach to duration of therapy, for pyelonephritis?

1. I use a fixed duration of therapy for most or all patients
2. I use a range of durations with a fixed maximum and minimum, and vary within that range according to patient or situation
3. I use a fixed minimum duration of therapy, which I extend in certain patients or situations (with no fixed maximum)
4. I use a fixed maximum duration of therapy, which I reduce in certain patients or situations (with no fixed minimum)
5. I have no fixed minimum or maximum duration, and treat in a manner entirely individualized to the patient or situation

The following question (4b) will vary depending on the answer provided in 4a.

If the respondent chooses “a” in response to 4a, then he/she is asked:

4b: What is the usual fixed duration of therapy you recommend for most or all patients?

Numbered selection from dropdown menu (1 – 28 days)

If the respondent chooses “b” in response to 4a, then he/she is asked:

4b: What is the fixed minimum and maximum duration of therapy you recommend for patients?

2 numbered selections from dropdown menu (1 – 28 days)

If the respondent chooses “c” in response to 4a, then he/she is asked:

4b: What is the fixed minimum duration of therapy you recommend for patients?

Numbered selection from dropdown menu (1 – 28 days)

If the respondent chooses “d” in response to 4a, then he/she is asked:

4b: What is the fixed maximum duration of therapy you recommend for patients?

Numbered selection from dropdown menu (1 – 28 days)

If the respondent chooses “e” in response to 4a, then he/she is asked:

4b: What is the most common duration of therapy you recommend for patients?

Numbered selection from dropdown menu (1 – 28 days)

4c. Would any of the following factors, if present, cause you to increase the duration of therapy proposed? (Check all that apply)

1. Comorbidities that alter immune function (e.g., Diabetes mellitus, chronic liver disease, chronic kidney disease)
2. Immunocompromised status (e.g. hematological malignancy, recent solid organ or hematopoietic stem cell transplantation, or receipt of immunosuppressive medications)
3. Clinically severe disease at presentation
4. Ongoing clinical symptoms/signs of inflammation at the site of infection
5. Persistent hypotension
6. Persistent fever
7. Persistent leukocytosis
8. Persistent biomarker of inflammation elevation (e.g., CRP, PCT, ESR)
9. None of the factors would result in increases to the duration of therapy proposed

4d. Please indicate the duration of treatment you would recommend in the following scenarios (assume each scenario provided below is independent of the others):

The patient had persistent nausea and vomiting >72 hours following initiation of treatment.

Numbered selection from dropdown menu (1 – 28 days)

Other _____ days

The *Proteus mirabilis* was classified as an ESBL

Numbered selection from a dropdown menu (1 – 28 days)

Other _____ days

The *Proteus mirabilis* in her cultures was not susceptible to Meropenem and other carbapenems

Numbered selection from a dropdown menu (1 – 28 days)

Other: ______ days

*Pseudomonas aeruginosa* was isolated from cultures instead of *Proteus mirabilis*.

Numbered selection from dropdown menu (1 – 28 days)

Other: ______ days

*Staphylococcus saprophyticus* was isolated from cultures instead of *Proteus mirabilis*.

Numbered selection from dropdown menu (1 – 28 days)

Other: ______ days

**Case Scenario #5**

You are seeing a 50-year-old female admitted with *E. coli* bacteremia of an unknown source; urine cultures taken prior to antibiotics and imaging are negative.

5a. What is your preferred approach to duration of therapy, for bacteremia of unknown origin?

1. I use a fixed duration of therapy for most or all patients
2. I use a range of durations with a fixed maximum and minimum, and vary within that range according to patient or situation
3. I use a fixed minimum duration of therapy, which I extend in certain patients or situations (with no fixed maximum)
4. I use a fixed maximum duration of therapy, which I reduce in certain patients or situations (with no fixed minimum)
5. I have no fixed minimum or maximum duration, and treat in a manner entirely individualized to the patient or situation

The following question (5b) will vary depending on the answer provided in 5a.

If the respondent chooses “a” in response to 5a, then he/she is asked:

5b: What is the usual fixed duration of therapy you recommend for most or all patients?

Numbered selection from dropdown menu (1 – 28 days)

If the respondent chooses “b” in response to 5a, then he/she is asked:

5b: What is the fixed minimum and maximum duration of therapy you recommend for patients?

2 numbered selections from dropdown menu (1 – 28 days)

If the respondent chooses “c” in response to 5a, then he/she is asked:

5b: What is the fixed minimum duration of therapy you recommend for patients?

Numbered selection from dropdown menu (1 – 28 days)

If the respondent chooses “d” in response to 5a, then he/she is asked:

5b: What is the fixed maximum duration of therapy you recommend for patients?

Numbered selection from dropdown menu (1 – 28 days)

If the respondent chooses “e” in response to 5a, then he/she is asked:

5b: What is the most common duration of therapy you recommend for patients?

Numbered selection from dropdown menu (1 – 28 days)

5c. When recommending a duration of therapy, how do you define “Day 1” of treatment in Gram negative bacteremia?

1. I start “day 1” of therapy from the first day of receipt of antibiotics
2. I start “day 1” of therapy from the first day of the definitive antibiotic therapy provided
3. I start “day 1” of therapy from the first day of negative blood cultures
4. I start “day 1” of therapy from the day of source control, if applicable
5. I start “day 1” of therapy from the day of clinical stability
6. Other, please specify: _______

5d. Using the case presented, please indicate your recommended duration of therapy in the following situations:

Blood cultures grew *Pseudomonas aeruginosa* instead of *E. coli*

Numbered selection from dropdown menu (1 – 28 days)

Other: ______ days

Blood cultures grew *Enterococcus faecalis* instead of *E. coli*

Numbered selection from dropdown menu (1 – 28 days)

Other: ______ days

Blood cultures grew *Bacteroides fragilis* instead of *E. coli*

Numbered selection from dropdown menu (1 – 28 days)

Other: ______ days

5e. Would any of the following factors, if present, cause you to increase the duration of therapy proposed? (Check all that apply)

1. Comorbidities that alter immune function (e.g., Diabetes mellitus, chronic liver disease, chronic kidney disease)
2. Immunocompromised status (e.g. hematological malignancy, recent solid organ or hematopoietic stem cell transplantation, or receipt of immunosuppressive medications)
3. Clinically severe disease at presentation
4. Persistent clinical symptoms/signs of inflammation at the site of infection
5. Persistent hypotension
6. Persistent fever
7. Persistent leukocytosis
8. Persistent biomarker of inflammation elevation (e.g., CRP, PCT, ESR)
9. None of the factors would result in increases to the duration of therapy proposed

**Closing Questions**

1. In general, do you believe most patients with uncomplicated bacterial infections should receive the same standard duration of therapy?
2. Yes
3. No
4. Please expand your response to #1 regarding your thoughts and perspectives on standard duration of therapy (fixed) compared to individualized therapy for uncomplicated bacterial infections.
5. Do you have anything else you’d like to add that was not captured in this survey?

Supplementary Table 1: Respondent Characteristics Associated with the Use of Individualized versus Fully Fixed Duration of Treatment for Each Clinical Scenario (Univariable Analyses)

| **Variable** | **Respondent Characteristic** | **Frequency, n (%)** | **Partially or Fully Individualized Approach to Treatment, n (%)** | **Fully Fixed Approach to Treatment, n (%)** | **P-value** |
| --- | --- | --- | --- | --- | --- |
| **SSTI** | | | | | |
| Country | Canada | 95 (47.5) | 84 (45.9) | 11 (64.7) | 0.14 |
|  | Other | 105 (52.5) | 99 (54.1) | 6 (35.3) |  |
| Location | Academic | 148 (74.0) | 138 (75.4) | 10 (58.8) | 0.15 |
|  | Non-Academic | 52 (26.0) | 45 (24.6) | 7 (41.2) |  |
| Specialty | General Internal Medicine Physician | 5 (2.5) | 5 (2.7) | 0 (0.0) | 0.67 |
|  | General Internal Medicine Trainee | 2 (1.0) | 2 (1.1) | 0 (0.0) |  |
|  | Infectious Diseases +/- Medical Microbiology Trainee | 22 (11.0) | 22 (12.0) | 0 (0.0) |  |
|  | Infectious Diseases Pharmacist | 6 (3.0) | 5 (2.7) | 1 (5.9) |  |
|  | Infectious Diseases Physician | 120 (60.0) | 108 (59.0) | 12 (70.6) |  |
|  | Infectious Diseases Physician + Medical Microbiologist | 31 (15.5) | 28 (15.3) | 3 (17.7) |  |
|  | Medical Microbiologist | 5 (2.5) | 5 (2.73) | 0 (0) |  |
|  | Medical Microbiology Trainee | 1 (0.5) | 1 (0.6) | 0 (0) |  |
|  | Other | 8 (4.0) | 7 (3.8) | 1 (5.9) |  |
| **Community-acquired pneumonia** | | | | | |
| Country | Canada | 95 (47.5) | 57 (43.2) | 30 (57.7) | 0.08 |
|  | Other | 105 (52.5) | 75 (56.8) | 22 (42.3) |  |
| Location | Academic | 148 (74.0) | 99 (75.0) | 38 (73.1) | 0.79 |
|  | Non-Academic | 52 (26.0) | 33 (25.0) | 14 (26.9) |  |
| Specialty | General Internal Medicine Physician | 5 (2.5) | 4 (3.0) | 1 (1.9) | 0.14 |
|  | General Internal Medicine Trainee | 2 (1.0) | 2 (1.5) | 0 (0.0) |  |
|  | Infectious Diseases +/- Medical Microbiology Trainee | 22 (11.0) | 18 (13.6) | 1 (1.9) |  |
|  | Infectious Diseases Pharmacist | 6 (3.0) | 2 (1.5) | 2 (3.9) |  |
|  | Infectious Diseases Physician | 120 (60.0) | 78 (59.1) | 34 (65.4) |  |
|  | Infectious Diseases Physician + Medical Microbiologist | 31 (15.5) | 18 (13.6) | 12 (23.1) |  |
|  | Medical Microbiologist | 5 (2.5) | 4 (3.0) | 0 (0.0) |  |
|  | Medical Microbiology Trainee | 1 (0.5) | 1 (0.8) | 0 (0.0) |  |
|  | Other | 8 (4) | 5 (3.8) | 2 (3.9) |  |
| **Acute Cholangitis** | | | | | |
| Country | Canada | 95 (47.5) | 50 (42.7) | 36 (55.4) | 0.1 |
|  | Other | 105 (52.5) | 67 (57.3) | 29 (44.6) |  |
| Location | Academic | 148 (74.0) | 90 (76.9) | 45 (69.2) | 0.26 |
|  | Non-Academic | 52 (26) | 27 (23.1) | 20 (30.8) |  |
| Specialty | General Internal Medicine Physician | 5 (2.5) | 5 (4.3) | 0 (0.0) | 0.37 |
|  | General Internal Medicine Trainee | 2 (1.0) | 2 (1.7) | 0 (0.0) |  |
|  | Infectious Diseases +/- Medical Microbiology Trainee | 22 (11.0) | 13 (11.11) | 5 (7.69) |  |
|  | Infectious Diseases Pharmacist | 6 (3.0) | 3 (2.6) | 1 (1.5) |  |
|  | Infectious Diseases Physician | 120 (60.0) | 63 (53.9) | 48 (73.9) |  |
|  | Infectious Diseases Physician + Medical Microbiologist | 31 (15.5) | 22 (18.8) | 8 (12.3) |  |
|  | Medical Microbiologist | 5 (2.5) | 3 (2.6) | 1 (1.5) |  |
|  | Medical Microbiology Trainee | 1 (0.5) | 1 (0.9) | 0 (0.0) |  |
|  | Other | 8 (4.0) | 5 (4.3) | 2 (3.1) |  |
| **Pyelonephritis** | | | | | |
| Country | Canada | 95 (47.5) | 48 (42.1) | 38 (58.5) | 0.04 |
|  | Other | 105 (52.5) | 66 (57.9) | 27 (41.5) |  |
| Location | Academic | 148 (74.0) | 86 (75.4) | 46 (70.8) | 0.49 |
|  | Non-Academic | 52 (26.0) | 28 (24.6) | 19 (29.2) |  |
| Specialty | General Internal Medicine Physician | 5 (2.5) | 3 (2.6) | 2 (3.1) | 0.04 |
|  | General Internal Medicine Trainee | 2 (1.0) | 2 (1.8) | 0 (0.0) |  |
|  | Infectious Diseases +/- Medical Microbiology Trainee | 22 (11.0) | 14 (12.3) | 3 (4.6) |  |
|  | Infectious Diseases Pharmacist | 6 (3.0) | 0 (0.0) | 4 (6.2) |  |
|  | Infectious Diseases Physician | 120 (60.0) | 65 (57.0) | 44 (67.7) |  |
|  | Infectious Diseases Physician + Medical Microbiologist | 31 (15.5) | 19 (16.7) | 11 (16.9) |  |
|  | Medical Microbiologist | 5 (2.5) | 4 (3.5) | 0 (0.0) |  |
|  | Medical Microbiology Trainee | 1 (0.5) | 1 (0.9) | 0 (0.0) |  |
|  | Other | 8 (4.0) | 6 (5.3) | 1 (1.5) |  |
| **BSI of unknown origin** | | | | | |
| Country | Canada | 95 (47.5) | 56 (45.2) | 30 (56.6) | 0.16 |
|  | Other | 105 (52.5) | 68 (54.8) | 23 (43.4) |  |
| Location | Academic | 148 (74.0) | 92 (74.2) | 38 (71.7) | 0.73 |
|  | Non-Academic | 52 (26.0) | 32 (25.8) | 15 (28.3) |  |
| Specialty | General Internal Medicine Physician | 5 (2.5) | 5 (4.0) | 0 (0.0) | 0.79 |
|  | General Internal Medicine Trainee | 2 (1.0) | 2 (1.6) | 0 (0.0) |  |
|  | Infectious Diseases +/- Medical Microbiology Trainee | 22 (11.0) | 11 (8.9) | 5 (9.4) |  |
|  | Infectious Diseases Pharmacist | 6 (3.0) | 2 (1.6) | 2 (3.8) |  |
|  | Infectious Diseases Physician | 120 (60.0) | 72 (58.1) | 36 (67.9) |  |
|  | Infectious Diseases Physician + Medical Microbiologist | 31 (15.5) | 23 (18.6) | 7 (13.2) |  |
|  | Medical Microbiologist | 5 (2.5) | 3 (2.4) | 1 (1.9) |  |
|  | Medical Microbiology Trainee | 1 (0.5) | 1 (0.8) | 0 (0.0) |  |
|  | Other | 8 (4.0) | 5 (4.0) | 2 (3.8) |  |

**Supplemental Figure 1a. Respondent Characterization of Day 1 of Therapy - Cholangitis**

**Supplemental Figure 1b. Respondent Characterization of Day 1 of Therapy – BSI of Unknown Source**
